# Regulation of Dishevelled DEP domain swapping by conserved phosphorylation sites

**DOI:** 10.1073/pnas.2103258118

**Published:** 2021-06-21

**Authors:** Gonzalo J. Beitia, Trevor J. Rutherford, Stefan M. V. Freund, Hugh R. Pelham, Mariann Bienz, Melissa V. Gammons

**Affiliations:** ^a^Medical Research Council Laboratory of Molecular Biology, Cambridge Biomedical Campus, Cambridge, CB2 0QH, United Kingdom

**Keywords:** Wnt signaling, Dishevelled, phosphorylation, domain swapping

## Abstract

Wnt signals bind to Frizzled receptors to trigger canonical and noncanonical signaling responses that control cell fates during animal development and tissue homeostasis. All Wnt signals are relayed by the hub protein Dishevelled. During canonical (β-catenin–dependent) signaling, Dishevelled assembles signalosomes via dynamic head-to-tail polymerization of its Dishevelled and Axin (DIX) domain, which are cross-linked by its Dishevelled, Egl-10, and Pleckstrin (DEP) domain through a conformational switch from monomer to domain-swapped dimer. The domain-swapped conformation of DEP masks the site through which Dishevelled binds to Frizzled, implying that DEP domain swapping results in the detachment of Dishevelled from Frizzled. This would be incompatible with noncanonical Wnt signaling, which relies on long-term association between Dishevelled and Frizzled. It is therefore likely that DEP domain swapping is differentially regulated during canonical and noncanonical Wnt signaling. Here, we use NMR spectroscopy and cell-based assays to uncover intermolecular contacts in the DEP dimer that are essential for its stability and for Dishevelled function in relaying canonical Wnt signals. These contacts are mediated by an intrinsically structured sequence spanning a conserved phosphorylation site upstream of the DEP domain that serves to clamp down the swapped N-terminal α-helix onto the structural core of a reciprocal DEP molecule in the domain-swapped configuration. Mutations of this phosphorylation site and its cognate surface on the reciprocal DEP core attenuate DEP-dependent dimerization of Dishevelled and its canonical signaling activity in cells without impeding its binding to Frizzled. We propose that phosphorylation of this crucial residue could be employed to switch off canonical Wnt signaling.

## Significance

We report a Dishevelled structural clamp, a β-sheet containing a conserved phosphorylation site immediately upstream of the Dishevelled, Egl-10, and Pleckstrin (DEP) domain, contacting a cognate surface on its partner DEP domain to stabilize the domain-swapped dimer. DEP dimers are essential for Wnt/β-catenin signal transduction, but because the Frizzled receptor–binding surface of the DEP monomer is masked in the dimer, domain swapping may need to be attenuated to sustain prolonged Frizzled binding that is needed to transduce noncanonical Wnt signals. Mutation of this phosphorylation site and, by implication, its phosphorylation destabilize DEP dimers, thereby disabling canonical signaling. We propose that the regulation of DEP domain swapping by phosphorylation provides a mechanism whereby Dishevelled could switch off canonical Wnt signals.

Wnt signaling cascades are ancient cell communication pathways that regulate cell fates during embryonic development and tissue homeostasis (1, 2). Extracellular Wnt ligands bind to seven-pass transmembrane Frizzled receptors and transduce signals to downstream effectors through Dishevelled, an intracellular hub protein that binds to Frizzled and assembles dynamic signaling complexes termed “signalosomes” (1, 3). Dishevelled pivots between alternative Wnt signaling branches to specify distinct outcomes (4). These branches are broadly defined as canonical (or β-catenin–dependent), typically driving cellular proliferation or differentiation (5, 6), and noncanonical, comprising a collection of signaling branches that coordinate cellular properties such as planar cell polarity (PCP) (7) and morphogenetic processes such as convergent extension (8, 9).

**Fig. 1.**
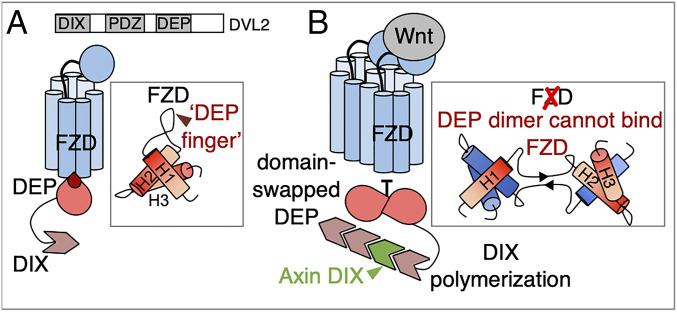
Cartoon of DVL domain architecture and function in canonical Wnt signaling. (*A*) Dishevelled with its three well-conserved domains, DIX, PDZ, and DEP. The DEP domain binds Frizzled directly through its prominent “DEP finger,” formed from the hinge loop separating H1 and H2 in the monomeric configuration. (*B*) Wnt signals cause DEP to dimerize by domain swapping. During this process, H1 from one DEP molecule is exchanged with H1 from another through extension of the hinge loop. As a consequence, the “DEP finger” undergoes a conformational change, resulting in a structurally distinct β-sheet connecting the two DEP molecules, which cannot bind to Frizzed. Domain swapping therefore promotes detaching of Dishevelled from the receptor complex.

Dishevelled has three well-conserved domains: an N-terminal Dishevelled and Axin (DIX) domain; central Postsynaptic density protein-95, Disk large tumor suppressor, Zonula occludens-1 (PDZ) domain; and C-terminal Dishevelled, Egl-10, and Pleckstrin (DEP) domain. Dishevelled is recruited to Frizzled by the DEP domain and assembles Wnt signalosomes via self-association of both its DIX and DEP domains (Fig. 1 *A* and *B*). The DIX domain undergoes reversible head-to-tail polymerization (10) to generate dynamic filaments of Dishevelled that are stably cross-linked by dimerization through the DEP domain (11). This rapidly increases the local concentration of Dishevelled and boosts its avidity for low-affinity effectors such as Axin, enabling Dishevelled to interact with these effectors even if present at a low cellular concentration (1, 3, 12).

**Fig. 2.**
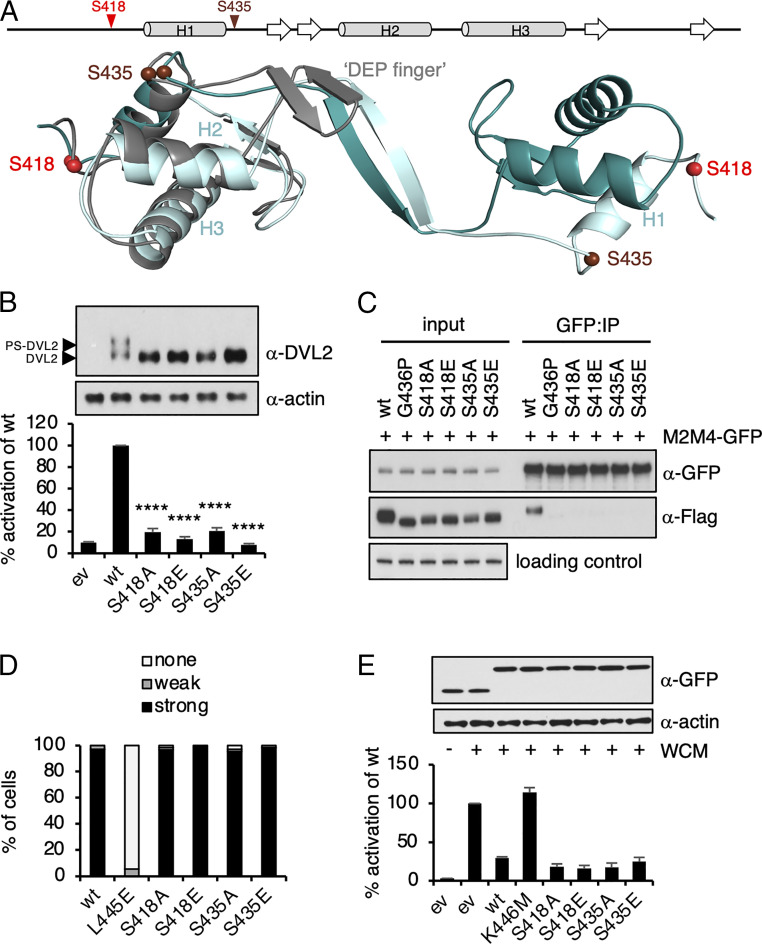
Mutations attenuating DEP dimerization and DVL2-dependent signaling. (*A*) Structure of the DEP dimer showing domain swapping (molecule A, dark turquoise; molecule B, light turquoise) superimposed on the DEP monomer (gray, “DEP finger”). S418 and S435 are shown (balls). (*B*) SuperTOP assays of HEK293T cells, expressing wt or mutant FLAG-DVL2 (as indicated; above, corresponding Western blot); ev, empty vector control; error bars, SEM of >3 independent experiments. (*C*) Western blots of immunoprecipitants (IPs) of polymerization-deficient DVL2 (M2M4-GFP) after coexpression with wt or DEP-mutant FLAG-DVL2 in transiently transfected HEK293T cells, probed with antibodies as indicated on the right; M2M4 was used instead of wt DVL2 to guard against confounding effects of DIX-dependent polymerization on DEP-dependent coIP (11). (*D*) Quantitative analysis of SNAP-FZD5-dependent recruitment of wt or mutant DEP-GFP to the plasma membrane (*n* = 100 cells scored in each case). (*E*) SuperTOP assays of HEK293T cells, monitoring the blocking of endogenous signaling in response to Wnt3a stimulation by overexpressed wt or mutant DEP-GFP (as indicated; above, corresponding Western blot); ev, GFP control; WCM, Wnt3a-conditioned medium (applied 6 h before lysis); error bars, SEM of >3 independent experiments. ****P < 0.0001; one-way ANOVA with repeated measures.

The DEP domain is a small globular domain composed of three α-helices and a flexible hinge loop between the first (H1) and second helix (H2), which, in the monomeric configuration, folds back on itself to form a prominent “DEP finger” that is responsible for binding to Frizzled (Fig. 1*A*) (13). DEP dimerization involves a highly unusual mechanism called “domain swapping” (14). During this process, H1 of one DEP monomer is exchanged with H1 from a reciprocal one through outward motions of the hinge loops, replacing intra- with intermolecular contacts. This results in a dimer whose DEP cores, almost identical in structure to the monomer, are connected by a β-sheet formed between the two hinge loops (Fig. 2*A*) (11). In other words, these hinge loops, which form the “DEP finger” in the monomer, undergo a major conformational change engaging in new intermolecular interactions that are likely to stabilize the dimeric configuration. Functional assays in Dishevelled null-mutant cells based on structure-designed mutants have revealed that this mechanism is essential for Wnt/β-catenin signaling (15). There are several examples of domain swapping underlying pathological processes (e.g., in neurodegenerative disease); however, the DEP domain of Dishevelled represents a rare example of a domain undergoing physiologically relevant domain swapping (16).

A consequence of domain swapping is that the Frizzled binding “DEP finger” undergoes a conformational change that is structurally incompatible with Frizzled binding (Fig. 1*B*) (13). Therefore, domain swapping blocks binding between DEP and Frizzled, which could cause detachment of Dishevelled from Frizzled. In turn, this would terminate Wnt signal transduction, as the signal relay depends on continued association between Frizzled and Dishevelled. For example, PCP signaling in *Drosophila* requires apical recruitment of Dishevelled (Dsh) by Frizzled to be maintained for many hours, even days, in stable, membrane-localized signalosomes that are clearly visible by confocal microscopy (17, 18). In contrast, Wnt/β-catenin signaling appears to need only a transient association between Frizzled and Dsh. This is illustrated by *dsh*^*1*^ flies that bear a mutation in the DEP finger (K417M), which reduces the membrane localization of Dishevelled and thus causes PCP defects without apparently affecting canonical Wnt signaling (7, 19, 20). The same mutation in Dvl2 (K446M) causes PCP and convergent extension defects when introduced into *Dvl1*^−/−^ *Dvl2*^−/−^ transgenic mice (21). Thus, either DEP domain swapping needs to be attenuated in PCP signalosomes or, if domain-swapped Dishevelled molecules were to remain within signalosomes, adaptor proteins would be required to mediate continued association between Dishevelled and Frizzled receptor complexes.

One mechanism by which the DEP domain could be regulated is by Wnt-induced phosphorylation (19, 20, 22), which accompanies the “activation” of Dishevelled (23, 24). During PCP signaling in flies, phosphorylation of Dishevelled correlates with its membrane recruitment by Frizzled (19, 20). Furthermore, its phosphorylation by Discs Overgrown (Dco), the *Drosophila* casein kinase-1ε (CK1ε) ortholog, promotes asymmetric localization of Dishevelled along the apical plasma membrane (25) and its stable association with junctional complexes (26). Dishevelled is phosphorylated on numerous serine (Ser) and threonine (Thr) residues, but it is unclear which of these are biologically important, as the vast majority of phosphorylations detected by mass spectrometry (MS) are not required for function or are functionally redundant (22, 25, 27, 28). However, we previously reported that single point mutations of two conserved Ser residues in the DEP domain (S418 and S435) reduce Wnt/β-catenin signaling (11) (*SI Appendix*, Fig. S1), suggesting that the phosphorylation of either Ser could regulate DEP domain function. Importantly, S418 is clearly a substrate for phosphorylation since high-throughput analysis of phosphorylation sites in breast cancer samples detected S418 phosphorylation by MS (29) (PhosphoSite). In addition, a recent comparative analysis of human Dishevelled-3 (DVL3) phosphorylation revealed that several Ser/Thr kinases are capable of phosphorylating S407 (the equivalent of DVL2 S418) in cells (30). Whether S435 is a bona fide substrate for phosphorylation remains to be determined; however, phosphorylation of its equivalent in flies, S406, was detected by MS in cells undergoing PCP signaling (28). Based on this evidence, we decided to investigate whether these or any other phospho-sites in the DEP domain affect signaling by altering DEP domain swapping.

Here, we show that these two highly conserved Ser residues (S418 and S435) are required for the stability of the DVL2 domain-swapped DEP dimer. We used NMR spectroscopy to demonstrate that S418, located immediately upstream of H1, engages in crucial noncovalent interactions with a structured loop that connects H2 and H3. As a consequence, a β-sheet forms that clamps down each H1 onto its reciprocal DEP core within a domain-swapped dimer, thereby providing stability to this DEP dimer without, however, affecting the binding between DEP monomer and Frizzled. An important corollary is that the phosphorylation of the key residue S418 within this clamp, for example, during noncanonical Wnt signaling, attenuates domain swapping, thereby allowing a stable association between DEP monomers and Frizzled. Our work has uncovered a pivotal residue within Dishevelled that negatively regulates DEP domain swapping, thereby antagonizing canonical Wnt signaling.

## Results

### Two Conserved Putative Phosphorylation Sites Are Required for DEP Dimerization and Canonical Wnt Signaling.

We previously reported that mutation of two Ser residues in the DEP domain reduced canonical signaling, as measured by a β-catenin–dependent T cell factor (TCF) transcriptional reporter (called SuperTOP) in signaling assays based on transient overexpression of FLAG-tagged DVL2 (FLAG-DVL2) in HEK293T cells (31). Based on these results, we decided to test whether other phosphorylatable residues in the DEP domain were required for DVL2-dependent signaling. We thus performed alanine (Ala)-scanning mutagenesis of all 11 Ser or Thr residues in the DEP domain, which revealed that only S418A and S435A significantly reduced DVL2-dependent SuperTOP activity by ∼80% compared to wild type (wt) (*SI Appendix*, Fig. S2). Note that a construct bearing a deletion of the conserved phosphorylation cluster upstream of DEP (amino acids 396 to 407 in human DVL2) still activated SuperTOP (*SI Appendix*, Fig. S2). Next, we mutated S418 and S435 to glutamic acid (Glu) to mimic the bulky negative charge of a phosphoserine and compared them to Ala substitutions. Transient overexpression of wt FLAG-DVL2 activated SuperTOP and promoted substantial phosphorylation of FLAG-DVL2 (detectable as multiple upward-shifting bands of FLAG-DVL2 on SDS-PAGE; to be called phospho-shifted DVL2, PS-DVL2) (Fig. 2*B* and *SI Appendix*, Fig. S2), which correlates with DVL2-dependent signaling (20, 22) and polymerization (10, 11). However, neither Glu nor Ala substitutions stimulated signaling or formed PS-DVL2 (Fig. 2*B*). These results are consistent with a previous study showing that mutations of DVL3 S407 (corresponding to DVL2 S418) reduced signaling by DVL3 and its condensation into supermolecular assemblies (32).

Overexpression of DVL2 enables it to signal independently of Wnt3a and of its recruitment to Frizzled at the plasma membrane, but signaling by overexpressed DVL2 nevertheless depends on DIX-dependent polymerization and DEP-dependent domain swapping (11). We therefore wondered whether the inactivity of our Ser mutants, located either side of H1 (Fig. 2*A*), might be due to a defect in DEP domain swapping. To test this, we used a coimmunoprecipitation (coIP) assay, based on differently tagged DVL2, to monitor DEP domain swapping–dependent dimerization of DVL2 (11). Indeed, either Ser mutant abolished coIP, similarly to the previously characterized. domain swapping mutant G436P (Fig. 2*C*) (11), which confirmed that these Ser residues are required for DEP domain swapping.

The minimal DEP domain is efficiently recruited to the plasma membrane by various Frizzled paralogs (e.g., FZD5) upon co-overexpression in cells, and this depends on its prominent protruding loop called DEP finger (11, 33, 34). Based on their location in the monomeric DEP structure, we anticipated that neither Ser would be required for FZD5-dependent DEP translocation to the plasma membrane, which was indeed the case; neither mutant affected recruitment of DEP tagged with GFP (DEP-GFP) to SNAP-FZD5 (Fig. 2*D* and *SI Appendix*, Fig. S2) nor its ability to block endogenous signaling in response to Wnt3a stimulation (Fig. 2*E*), unlike mutants that affect the binding between the DEP finger and Frizzled (L445E and K446M) (11, 33, 34). This confirms that these two Ser residues are dedicated to the dimerization of DEP rather than its binding to Frizzled as a monomer.

### S418 Mutations Reduce the Stability of the DEP Domain.

**Fig. 3.**
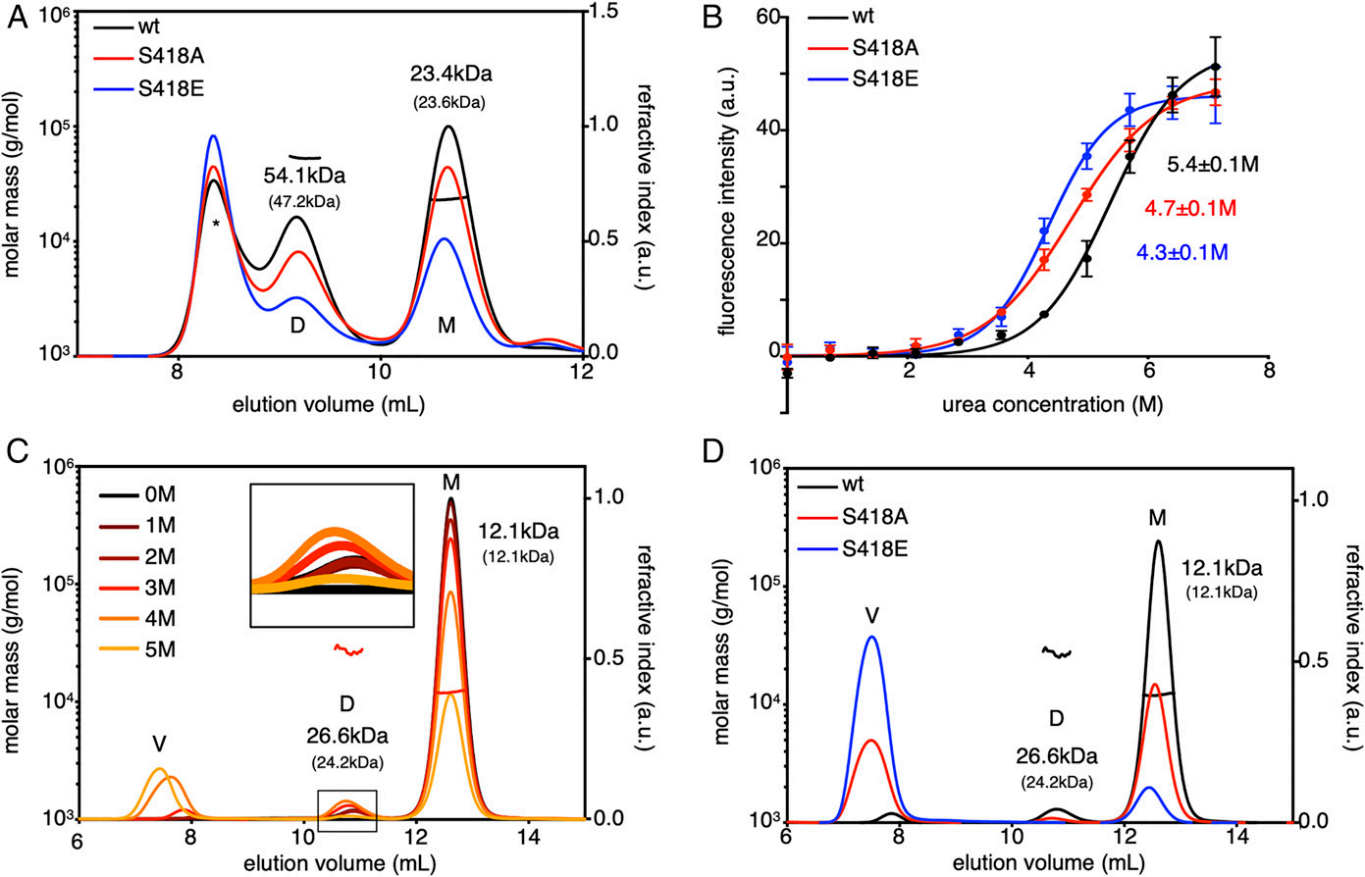
SEC-MALS and thermodynamic analysis of purified DEP domain. (*A*) Elution profiles of unfractionated wt (black) or mutant (S418A, red; S418E, blue) Lip-DEP_402__to__510_ revealing monomers (M), dimers (D), and higher molecular mass species (*); molecular mass (MM) for M and D were calculated (expected MM shown below in brackets). (*B*) Urea-induced denaturation equilibrium curves following 8-h incubations with urea at room temperature; EC_50_ ± SEM of three independent experiments (labeled). (*C*) Elution profiles of cleaved wt DEP monomer following incubation with urea (0 M, black*;* 1 to 5 M, color) revealing M, D, and V. MM of M and D were determined at 3 M urea (expected MM shown below in brackets, as in *A*). (*D*) Elution profiles of wt (black) and mutant (S418A, red; S418E, blue) DEP monomer following incubation with 3 M urea (M, D, V, and MM as in *C*).

Next, we assessed our Ser mutants in vitro by size-exclusion chromatography with multiangle light scattering (SEC-MALS) of purified Lipoyl-tagged DEP (Lip-DEP_402–510_). For simplicity, only the results for S418A and S418E are shown, but S435A and S435E essentially behaved the same. SEC-MALS profiles revealed that the mutant domains produce significantly fewer dimers compared to wt Lip-DEP_402–510_ (Fig. 3*A*), as previously reported for other DEP domain swapping mutants (11). To determine whether mutant DEP reduced protein stability, we used urea-induced equilibrium denaturation to monitor unfolding of wt and mutant DEP monomers (following cleavage of the Lip tag) by recording intrinsic florescence intensity. The urea half maximal effective concentration (EC_50_) of S418A and S418E were reduced compared to wt, consistent with a decrease in their conformational stability (Fig. 3*B*). In addition, the thermal stability of these mutants was also reduced if assayed by Prometheus, an instrument that detects unfolding by monitoring changes in intrinsic fluorescence intensity (i.e., each mutant unfolded at a lower temperature compared to wt DEP) (*SI Appendix*, Table S1).

Domain swapping requires either partial unfolding of swapped elements or complete unfolding of the whole domain (16, 35). Furthermore, interconversion between monomers and domain-swapped dimers is typically separated by high energy barriers and requires high protein concentration (16), and it is therefore challenging to observe these monomer-to-dimer interconversions under standard in vitro assay conditions. To examine the extent of DEP unfolding during domain swapping, we therefore used SEC-MALS following exposure of monomeric DEP to urea concentrations expected to induce partial protein unfolding (<EC_50_; 0 to 5 M urea). The resulting elution profiles revealed that a fraction of DEP monomer (∼5 to 10%) shifted to dimer at 2 to 4 M urea concentrations. We determined an optimal urea concentration of 3 M, as this induced dimer formation without causing significant unspecific aggregation (i.e., elution in the void volume), as observed at higher urea concentrations (5 M) that caused significant aggregation (Fig. 3*C*). Therefore, the DEP domain is stable and maintains most of its secondary structure under the conditions (3 M urea) that allow it to undergo domain swapping. However, if we apply the same conditions to S418A and S418E, each of these mutants aggregates and thus transitions from monomer to the void volume (Fig. 3*D*).

Our results using S418E and S435E mutants suggest that phosphorylation of either site would also reduce DEP stability. To test this directly, we attempted site-specific phosphorylation of the DEP domain in vitro. This is challenging because kinases are generally promiscuous and lack the specificity needed to selectively phosphorylate individual sites. Therefore, we used a technology based on an orthogonal SepRS/transfer RNA (tRNA)^pSer^_CUA_ aminoacyl-tRNA synthetase/tRNA pair to direct site-specific incorporation of phosphoserine (pSer) during synthesis of recombinant proteins in *Escherichia coli* (36). As our S418E and S435E mutants essentially behaved the same, we opted to introduce pSer at S435 because incorporation at sites close to the N terminus tend to be rather inefficient. However, because of low yields, we were unable to carry out additional purification steps such as removing the Lip tag. Therefore, we first tested wt Lip-DEP by SEC-MALS following exposure to urea concentrations (as was done for cleaved DEP; Fig. 3*C*) and determined 3.5 M as optimal to induce domain swapping without causing aggregation (*SI Appendix*, Fig. S3). Using these conditions, we also confirmed that Lip-DEP S435A and S435E mutants transitioned to the void volume (*SI Appendix*, Fig. S3), as was observed for cleaved equivalents (S435 substitutions behaved the same as S418 substitutions shown in Fig. 3*D*).

During purification, we used anion exchange chromatography (AEC) to enrich for pSer (Lip-DEP S435pS) and the remaining protein fraction as an unphosphorylated internal control (Lip-DEP ctrl). MS analysis revealed that more than half (60.4%) of the total S435 was phosphorylated (S435pS). The rest of the protein contained unphosphorylated Ser (8.6%) or misincorporation of glutamine (Gln, 15.8%) and tyrosine (Tyr, 13.5%) (*SI Appendix*, Fig. S4). Note that misincorporation of Gln and Tyr was also observed in the internal control (79.3% and 9.2%, respectively). Therefore, we tested both samples (S435pS and ctrl) alongside Lip-DEP wt in SEC-MALS following exposure to 3.5 M urea. Like our Glu substitutions, S435pS aggregates and transitions to the void volume (*SI Appendix*, Figs. S3 and S4). The internal control (which is predominantly S435Q, ∼80%) also partially transitioned in the void (*SI Appendix*, Fig. S4). This suggests that S435pS or Gln substitution behave like Glu and Ala mutants in destabilizing the DEP, although this was not tested directly. The same proportion of domain swapping is observed in all three samples, suggesting that dimerization in this case does not reflect incorporation of pSer but misincorporation of other amino acids that are permissive for domain swapping (*SI Appendix*, Fig. S4). However, this interpretation, while plausible, remains speculative, as we were unable to determine the proportion of S435 misincorporation for each eluted peak following SEC-MALS (monomer, dimer, and void). Despite this caveat that we were unable to generate pure DEP-S435pS in vitro*,* given that the predominant species within our sample was DEP exclusively phosphorylated at S435, our results are consistent with the notion that pSer incorporation at S435, and thus phosphorylation of S435, destabilizes the DEP domain. We anticipate that the same would be true if pSer were incorporated at S418, given the similar behavior of S418 and S435 mutants (Fig. 3*D* and *SI Appendix*, Fig. S3). We conclude that S418E and S435E substitutions are valid surrogates for phosphorylation, justifying the use of these phosphomimetic mutants for further analysis. Their increased sensitivity to urea is consistent with the notion that these mutant domains are less stable than wt DEP, mostly likely because of a loss of stabilizing noncovalent bonds.

### Stabilization of the DEP Fold by Intramolecular Contacts between S418 and DEP Core.

**Fig. 4.**
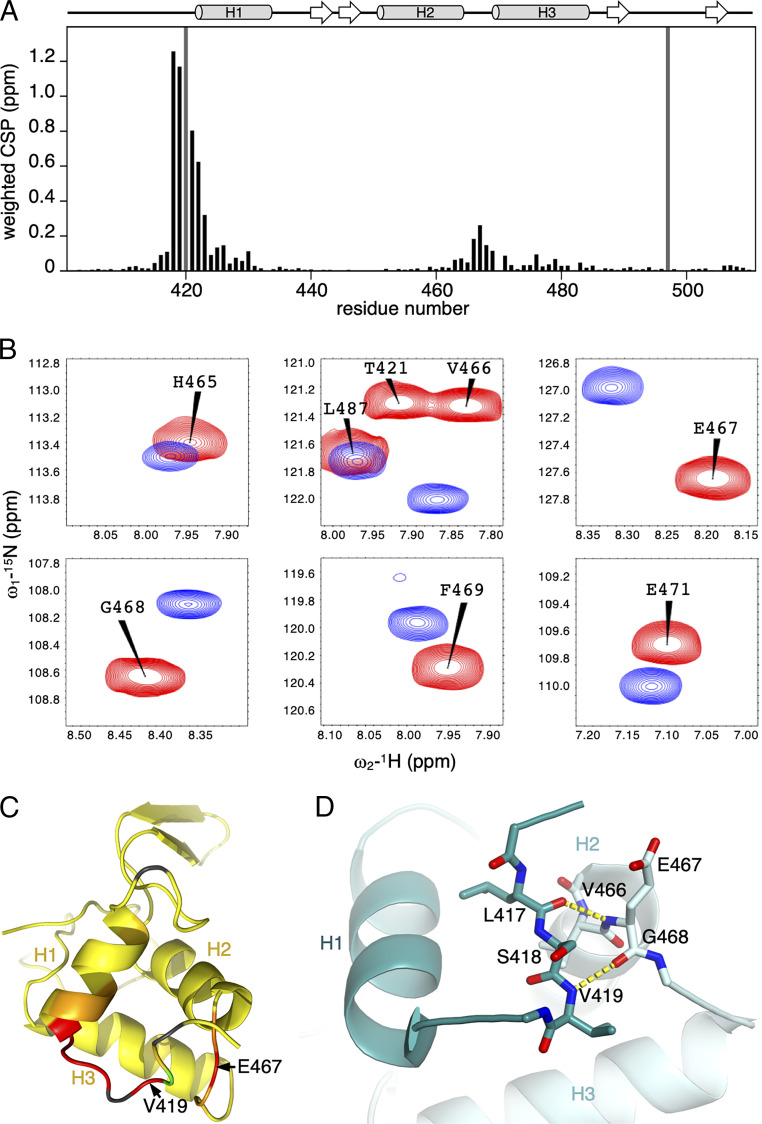
Intramolecular contacts by S418 as revealed by NMR. (*A*) Quantification of CSP for each residue caused by S418E (black bars); two residues were unassigned (gray bars); above, secondary structure representation. (*B*) Overlay of ^1^H-^15^N HSQC spectrum from wt (red) and S418E (blue) for a selection of residues exhibiting CSPs. (*C*) Heatmap of CSPs projected onto the solution monomer structure of mouse Dvl1 DEP (PDB 1FSH). (*D*) Structure of the DVL2 DEP dimer (PDB 5SUZ), revealing hydrogen bonds typical of a short parallel β-sheet; secondary structure elements are labeled for molecule A (dark turquoise), and molecule B (light turquoise); key residues are in stick (red, oxygen; blue, nitrogen); yellow dashed lines, hydrogen bonds.

In order to discover these putative contacts between S418 and the DEP domain, we used NMR to probe the structural environment of the DEP monomer in solution, focusing on the S418E phosphomimetic mutant. We chose S418 because it is a known substrate for phosphorylation and because it is highly unlikely that S418E would act by merely altering the flexibility of the hinge loop. A ^1^H-^15^N heteronuclear single-quantum coherence (HSQC) spectrum of ^15^N-labeled monomeric S418E was overlayed with a reference HSQC spectrum from wt DEP [whose resonances have been assigned previously (11)]. S418E produced well-dispersed HSQC spectra (*SI Appendix*, Fig. S5), consistent with stable folded protein, and highly conservative chemical shift perturbations (CSPs) relative to wt, indicating that its overall fold is highly similar to that of wt DEP. Most CSPs were localized either upstream of H1 or in a well-conserved region connecting H2 and H3 (Fig. 4*A*), encompassing residues V466, E467, and G468 (Fig. 4*B*). A “heatmap” of CSPs projected onto the structure of monomeric mouse Dvl1 DEP [Protein Data Bank (PDB) 1FSH (13)] confirms that S418 binds to the region connecting H2 and H3 (Fig. 4*C*). As the relative configurations of the α-helices are the same in the monomer and domain-swapped dimer, we expected the same interface to form in the domain-swapped conformation. Indeed, the high-resolution DVL2 domain-swapped crystal structure (PBD: 5SUZ; ∼2 Å) shows hydrogen bond patterning between E467 and L417 as well as V419, consistent with a classical short parallel β-sheet (hydrogen bond = 2.9 Å; Fig. 4*D*).

### A Crucial Role of S418 in Conferring DEP Domain Stability.

**Fig. 5.**
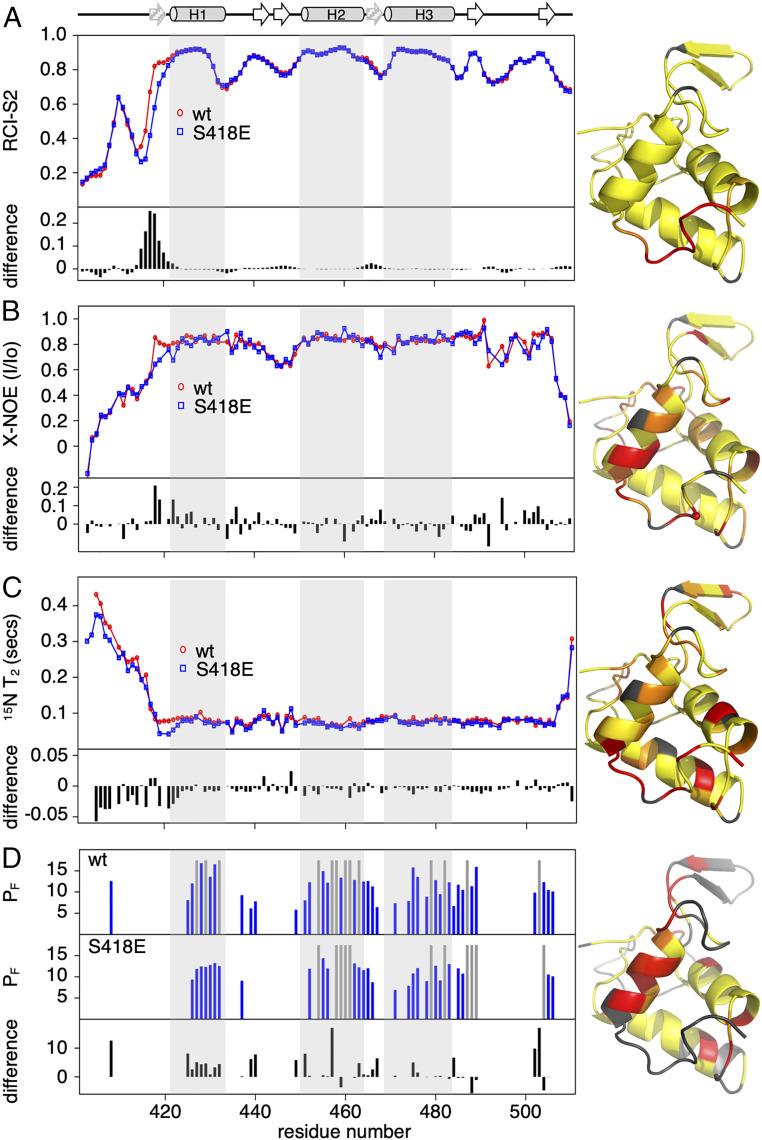
Conformational dynamics of DEP. RCI-S^2^ order parameters generated using the program TALOS+ (*A*) (37), ^15^N relaxation measurement {^1^H}^15^N-NOE (*B*) and ^15^N relaxation time-constant T_2,_ (*C*) BEST-TROSY H-D exchange time course (*D*), used to generate single residue P_F_ for wt (*Top*) and S418E (*Middle*). Several residues failed to exchange during the experiment (gray bars). (*A*–*D*) Differences between wt (red) and S418E (blue) shown underneath each panel and after projection onto heat maps (PDB 1FSH); gray, unassigned residues (*A*–*C*) or unprotected residues (*D*). (*Top*) Schematic representation of DEP structural elements including previously unknown β-strands inferred from our results (gray arrows); (*Bottom)* residue numbers.

Having obtained backbone resonance assignments for both wt and S418E mutant, we assessed the backbone conformational changes that can be inferred from the random coil index (RCI) chemical shift order parameters using the program TALOSe+ (37) (Fig. 5*A*). RCI-S^2^ order parameters were almost identical for wt and mutant throughout the entire sequence except for the region between R415 and D422 at the start of H1. Upstream of H1, the wt protein shows backbone order parameters similar to the bulk of the structured core up to and including S418. Residues to the N terminus of S418 are disordered. However, in the mutant, the less ordered region includes all residues upstream of H1, revealing that S418E disfavors the formation of the contacts between S418 and the structured region connecting H2 and H3. Loss of these contacts is evidently sufficient to destabilize the packing of the domain-swapped element and to inhibit formation of stable homodimers (Figs. 2*B* and 3*A*).

The apparent changes in backbone dynamics of the monomeric DEP N terminus were corroborated from ^15^N relaxation measurements, specifically, steady-state amide {^1^H}^15^N heteronuclear nuclear Overhauser effects (hetNOE) and the ^15^N relaxation time-constant *T*_2_. The relaxation measurements revealed that the region N-terminal to H1 is more flexible in the mutant as indicated by several hetNOE values below 0.7 (S418, V419, and D422; Fig. 5*B*). The backbone remains structured up to the S418 contact site in wt DEP but is disordered upstream of D422 in the mutant. Consistent with this, residues mapping to this region show reduced ^15^N *T*_2_ values in wt S418E, reflecting an increased conformational exchange contribution to relaxation (Fig. 5*C*). We also examined how the destabilizing S418E mutation affects amide backbone hydrogen/deuterium exchange protection factors (P_F_). A BEST-TROSY H–D exchange time course was recorded to compare slow exchange rates (10^−3^ to 10^−7^ ⋅ s^−1^) for both wt and S418E proteins to single-residue resolution. As expected, the location of highly protected amide groups reflects the hydrogen bonds that stabilize the secondary and tertiary protein structure but does not include the structured region upstream of H1, showing amide hydrogens in this region exchange with solvent during the experiment dead time (Fig. 5 *D* and *A*). Differences in protection between wt and mutant were minor (reflecting the well-dispersed HSQC mutant spectrum); however, a significant number of residues were consistently less protected in the S418E mutant. Notably, the E467 amide hydrogen in the H2-H3 linker, which is H-bonded to L417 in the wt crystal structures, shows a strong P_F_ in wt DEP but exchanged within the experiment dead time for the mutant. This is consistent with loss of the H-bonded contact inferred from the backbone dynamics data.

We projected P_F_ differences for residues more exposed in the mutant onto the monomeric structure to generate a heatmap. This highlights that the greatest differences are observed for residues likely involved in stabilizing loop and helix contacts, while few differences were observed in key contacts in the hydrophobic core (Fig. 5*D*). We also assessed fast-exchange rates on the millisecond-to-second timescale, using phase-modulated CLEAN chemical exchange experiments; however, exchange on this timescale was only evident for amides in the unstructured N-terminal tail, whereas wt and mutant were identical. We conclude that wt DEP is structured upstream of H1 (up to and including S418) and forms dynamic contacts with the linker between H2–H3, thereby forming a β-sheet. Furthermore, the formation of this β-sheet is blocked by mutation of S418. Within the dimer, formation of this β-sheet is intermolecular and, therefore, necessary for stabilizing domain-swapped DEP.

### Intermolecular Contacts Mediated by S418 Are Necessary for DEP Domain Swapping and Canonical Wnt Signaling.

**Fig. 6.**
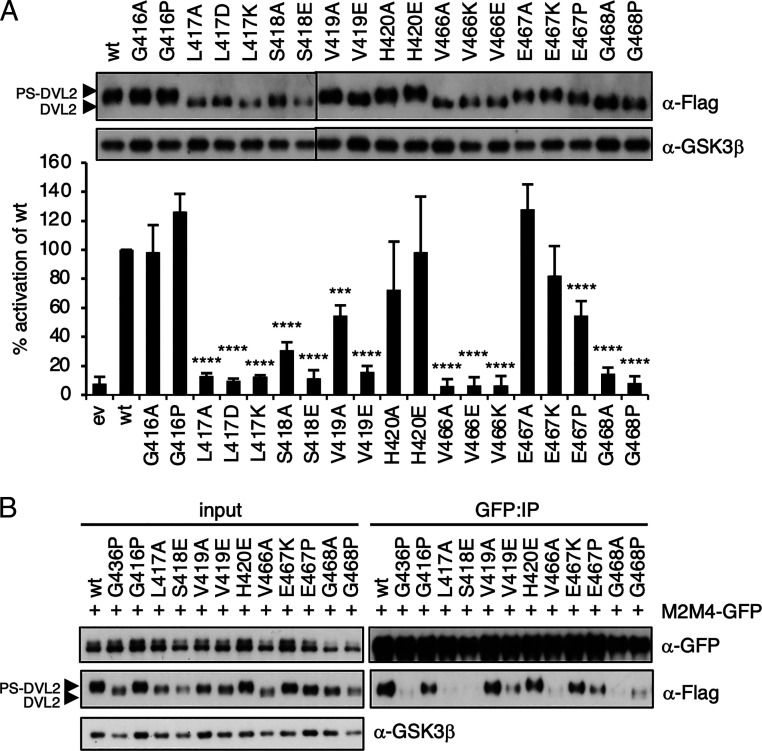
Intermolecular contacts necessary for DEP domain swapping. (*A*) SuperTOP assays of HEK293T cells expressing wt or mutant FLAG-DVL2 (as indicated; above, corresponding Western blot); ev, empty vector control; error bars, SEM of >3 independent experiments; one-way ANOVA with repeated measures; ****P < 0.0001, ***P < 0.001. (*B*) Western blots of IPs of M2M4-GFP after coexpression with wt or mutant FLAG-DVL2, as in Fig. 1*C*, probed with antibodies as indicated on the right. Note that in this experiment, M2M4-GFP expression was slightly reduced upon coexpression with G468A (lane 12) and G468P (lane 13).

Given that the ^1^H-^15^N HSQC spectra for S418E showed localized CSPs mapping to the structured region connecting H2–H3 (Fig. 4 *A*–*C*), we designed an additional 19 mutations to test their effect on Dishevelled signaling by FLAG-DVL2 overexpression in SuperTOP assays. Guided by the contacts visible in the domain-swapped DEP crystal structure (PDB: 5SUZ), we targeted residues surrounding S418 (G416 to H420) and the H2–H3 linker (V466 to G468; Fig. 6*A*). Mutation of L417 and V419, but not of the surrounding residues G416 and H420, behaved similarly to S418 in reducing SuperTOP activity (Fig. 6*A* and *SI Appendix*, Fig. S6). In addition to Glu and Ala substitutions, we also mutated S418 to other amino acids (summarized in *SI Appendix*, Table S2). Conservative replacements to Thr (often replacing Ser in orthologous proteins; *SI Appendix*, Fig. S1) and asparagine, which have polar uncharged side chains like Ser, were well tolerated and signaled normally, whereas leucine, glycine, and arginine mutants failed to do so. Although the L417 side chain projects away from the interface, its mutations can be rationalized by a loss of stabilizing hydrophobic interactions with T421, V426, A429, W461, and V466 (*SI Appendix*, Fig. S6). Based on our NMR results, we predicted that mutation of key contacts in the H2-H3 region such as V466 and G468 would abolish signaling, which was indeed the case (Fig. 6*A*). The side chain of E467 projects away from the interface, and so it was not surprising that mutation of E467 to Ala or lysine signaled normally. However, we also tested an E467P mutation (whereby the proline nitrogen backbone is not available for hydrogen bonding), which significantly attenuated SuperTOP (Fig. 6*A*). To confirm that these signaling-defective mutants attenuate domain swapping in vivo, we monitored DVL2 coIP for representative mutations of each residue (as in Fig. 2*B*). The strength of coIP correlated with signaling defects (i.e., the mutants that failed to signal also failed to coIP) (Fig. 6*B*). These results define the residues contributing to intermolecular contacts that are necessary for stable domain-swapped DEP and consequent signaling activity to β-catenin.

To confirm that these intermolecular contacts are required to transduce Wnt3a signals to β-catenin rather than for Wnt-independent signaling by overexpressed FLAG-DVL2, we employed our previously characterized Dishevelled complementation assay whereby DVL2-GFP is stably re-expressed at physiological levels in Dishevelled null cells (DVL triple knock out [TKO]), resulting in a strictly Wnt-dependent signaling activity of DVL2-GFP (15). We selected mild Ala substitutions of the strongest mutation upstream of H1 (S418A) and in the H2–H3 linker (V466A) to compare to wt DVL2-GFP. Neither mutant cell line activated SuperTOP in response to Wnt3a, unlike wt DVL2-GFP, which did so efficiently (*SI Appendix*, Fig. S7). Indeed, both mutants essentially behaved the same as previously reported domain swapping-defective mutants, G436P and E499G (15). We conclude that the intermolecular contacts, which stabilize the domain-swapped DEP domain, are essential for endogenous Dishevelled to transduce canonical Wnt signals to β-catenin.

## Discussion

Our work uncovered intermolecular contacts between reciprocal DEP monomers that stabilize the domain-swapped DEP dimer that are essential for canonical Wnt/β-catenin signaling by Dishevelled. These contacts are mediated by a deeply conserved phosphorylation site, and our results imply that the phosphorylation of this residue would block these contacts and thereby attenuate domain swapping of DEP and favor its monomeric state. Since DEP dimerization is essential for Wnt signaling to β-catenin, we propose that this phosphorylation event would attenuate canonical signaling but allow noncanonical signaling which requires long-term association of Dishevelled with Frizzled through its monomeric DEP domain. We hypothesize that phosphorylation of this key sensor residue upstream of DEP has the potential to regulate the switch between canonical and noncanoncial Wnt signaling. While our hypothesis is plausible and consistent with our data, its physiological relevance will require further investigation in tissues of whole animals, for example, during PCP signaling in *Drosophila*.

**Fig. 7.**
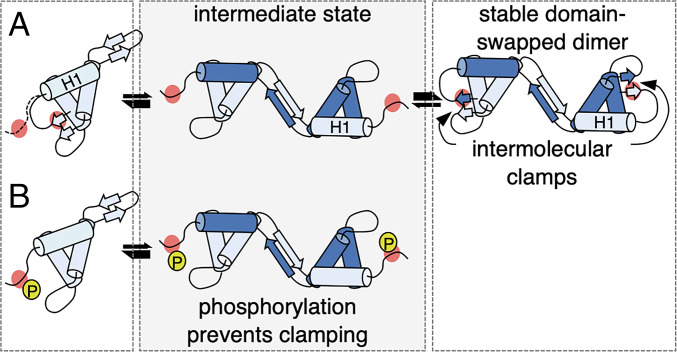
Model of phosphorylation-dependent control of DEP domain swapping. (*A*) DEP monomer (light turquoise) showing the S418 phospho-residue (red circle) undergoing dynamic exchange (dotted line) and forming intramolecular contacts. During domain swapping, DEP molecule A partially unfolds to donate its H1 to DEP molecule B (dark turquoise), adopting an intermediate conformational state. The transition to a stable DEP dimer requires intermolecular contacts mediated by S418 and its flanking residues to clamp down H1 from molecule A onto the hydrophobic core of molecule B. (*B*) Disruption of intramolecular contacts, for example by phosphorylation (*P*, yellow circle), does not affect the function of the DEP monomer in binding to Frizzled but attenuates the formation of a stable and functional domain-swapped DEP dimer.

The intermolecular contacts between the structured region upstream of H1 and the H2–H3 linker region are consistent with a classical short parallel β-sheet, which tethers or “clamps down” the swapped H1 from one DEP molecule to the hydrophobic core of another one (Fig. 7*A*). Our work with recombinant wt and mutant DEP has shown that this is essential for the stability of the DEP dimer in solution. Based on our structure-designed point mutations, we are able to predict S418 phosphorylation to block the clamping down of the swapped H1 on the reciprocal DEP molecule (because the bulky charge of a phosphoserine is expected to block or disrupt intermolecular contacts), thus rendering the dimer thermodynamically unstable and shifting the equilibrium in favor of its monomeric conformation (Fig. 7*B*). In other words, phosphorylation at this site would not be compatible with the formation of a stable DEP dimer. Our H–D exchange analysis revealed that the corresponding contacts in the monomer (formed in an intra- as opposed to intermolecular fashion) undergo dynamic exchange, or “conformational breathing,” in solution, which implies that their formation is not limiting for DEP monomer function despite being essential for dimer stability. A precedent for this was the discovery of a salt-bridge network of interactions that constrains the β3–β4 loop, which selectively stabilizes the hydrophobic DEP core in the dimeric conformation but appears dispensable for the function of DEP monomer in binding to Frizzled (11). Similarly, mutations of S418 (or, by implication, its phosphorylation) reduce the stability of the DEP domain and selectively inhibit the DEP dimer function without detectably affecting monomer function. Evidently, the domain-swapped DEP dimer is far more reliant than the DEP monomer on a number of weak intra- and intermolecular noncovalent contacts in terms of its stability and function.

Our evidence implicates the phosphorylation of S418, or its equivalent in Dishevelled paralogs (*SI Appendix*, Fig. S1), as a key event for the pivoting between canonical and noncanonical Wnt signaling. This residue clearly gets phosphorylated in vivo, although the physiological conditions required for its phosphorylation have not been determined. One of the kinases shown to phosphorylate this residue is PKCδ (30), which is required for convergent extension in *Xenopus* (38) (i.e., for noncanonical Wnt signal transduction). This supports the notion that the phosphorylation of this key DEP residue by PKCδ would allow Dishevelled to bind to Frizzled via monomeric DEP, consistent with the observation that PKCδ promotes the recruitment of Dishevelled to the plasma membrane and to Frizzled (38), whereby the activation of this kinase is essential for the transduction of noncanonical Wnt/Ca^2+^ signals (39). Similarly, the PKCζ isoform has been shown to act through the DEP domain to phosphorylate DVL3 and promote its stability (40).

Another candidate kinase in support of our model is CK1ε (41), which has been implicated in phosphorylating numerous Dishevelled residues. CK1ε binds to Dishevelled (41, 42) and, interestingly, blocks the formation of Dishevelled puncta in cells upon overexpression (41, 43), whereby the formation of these puncta depends on DEP domain swapping (11). Furthermore, mutation of *Drosophila* CK1ε (*dco*) reduces the phosphorylation of Dishevelled, blocks its asymmetric localization, and decreases its junctional instability, thereby causing PCP defects (26, 44). We note, however, that although the S418 phosphorylation site is well conserved throughout the animal kingdom, it does not appear to be present in *Drosophila* Dsh (*SI Appendix*, Fig. S1) but is replaced by an aspartic acid, which, based on our results, would be incompatible with intermolecular clamping down of its DEP. Yet Dsh DEP can substitute for DVL2 DEP in domain swapping assays in cells (11), which suggests that Dsh DEP utilizes other motifs upstream of H1 to form stabilizing intermolecular contacts during domain swapping. However, it is possible that DEP domain swapping in *Drosophila* is regulated via phosphorylation of the other conserved Ser residue, S406 (corresponding to S435 in human DVL2), located in the hinge-loop region (*SI Appendix*, Fig. S1), where phosphorylation has been detected, albeit sporadically, by MS in cells undergoing PCP signaling (28). Although these authors concluded that Ser/Thr phosphorylation of Dsh is not required for its function, several individual sites including S406 were not tested in the functional assays used in this study. Thus, a crucial role for this residue would have been missed. Whether S406/S435 is a bona fide substrate for phosphorylation during noncanonical Wnt signaling remains to be determined; however, our results clearly predict that its phosphorylation would attenuate DEP domain swapping, similarly to phosphorylation of S418, likely by altering the flexibility of the hinge region, a biophysical property that is well known to play a pivotal role in promoting domain swapping (16, 45).

We have proposed that the phosphorylation of two conserved phosphorylation sites in the DEP domain alters its conformational equilibrium to favor its monomeric state, thereby disallowing canonical signaling activity of Dishevelled and favoring transduction of noncanonical Wnt signals. According to the evidence available to date, noncanonical Wnt signal transduction relies on relatively stable, long-lived Wnt signalosomes, such as those observed during PCP signaling in wing imaginal discs (17, 18). However, it is conceivable that not all noncanonical signaling branches will require a stable association with Frizzled, and therefore, the need to attenuate domain swapping may not be universal to all noncanonical signals. Future studies are needed to test our hypothesis that the phosphorylation of the two conserved DEP phosphorylation sites uncovered by our work is pivotal for the switching of cells from canonical to noncanonical Wnt signaling.

## Materials and Methods

### Plasmids and Antibodies.

The following plasmids were used: human DVL2-GFP and FLAG-DVL2 (12); FLAG-DVL2, E499G, K446M (46), G436P, S418A, and S435A (11); and SNAP-FZD5 (47). Lip-DEP_402–510_ was megaprimer cloned into Lip-DEP_416–511_ (11). His_6_ N terminally tagged LipDEP_416–511_ (LipDEP_416–511_-His_6_) was cloned into a pNHD vector (36) with Gibson assembly, and an amber mutation (TAG) at position encoding for S435 was inserted by QuickChange cloning. DVL2 and DEP point mutations were generated by standard procedures and verified by sequencing. The following antibodies and resins were used: α-FLAG (Sigma) α-GFP (Sigma), α-actin (Abcam), α-SNAP (NEB), α-DVL2 (CST), and α-GSK3β (CST).

### Cell-Based Assays.

HEK293T cells were obtained from the European Collection of Cell Cultures (authenticated by short tandem repeat DNA profiling) and regularly tested for *Mycoplasma* infection. Cells were cultured and transfected and coIPs conducted essentially as described (46). Stable pBabe DVL2-GFP were generated in DVL TKO HEK293T cells essentially as described (15); in addition, control (wt; DVL2-GFP) stable cell lines were regenerated every time a new mutant was made for direct comparison. Single confocal images were acquired at identical settings with a Zeiss Confocal Microscope. For SuperTOP assays (31), HEK293T cells were lysed 16 h after transfection and analyzed with the Dual-Glo Luciferase Reporter Assay (Promega) according to the manufacturer's protocol. Values were normalized to Renilla luciferase and are shown as mean ± SEM expressed as percent activation of wt.

### Protein Purification, Thermal Stability, and Urea-Induced Denaturation.

Lip-DEP_402–510_ and Lip-DEP_416–511_ proteins were expressed in *E. coli* BL21-CodonPlus(DE3)-RIL cells (Stratagene) at 37 °C, induced with isopropyl β-D-1-thiogalactopyranoside (IPTG) at optical density (OD) 0.8, grown for 6 h at 24 °C and purified at 4 °C using NiNTA resin, followed immediately by gel filtration with S200 SEC Superdex 200 Increase 10/300 GL (SEC). Note that gel filtration was always performed on freshly prepped protein. The Lip tag was removed by tobacco etch virus protease for NMR, thermal stability, and urea-induced denaturation experiments. For protein synthesis with genetically encoded unnatural amino acids, Lip-DEP_416–511_-His_6_ was expressed in *E. coli* BL21*ΔSerB*(DE3) cells containing a pKW2 EF-Sep vector (36). Cells were grown at 37 °C, induced with IPTG at OD 0.8, and grown for 4 h at 37 °C. pSer was added to the media at 4 mM (half prior to starting expression and half after IPTG induction). Purification was done at 4 °C using an NiNTA 1-mL resin column (HisTrap FF Crude GE Healthcare), followed immediately by gel filtration with SEC and AEC with a HiTrap 5-mL Q HP. Phosphatase Inhibitor Mixture Tablets (PhosSTOP Roche) were used in all buffers. MS analysis was done by liquid chromatography (LC)-MS/MS (Ultimate U3000 HPLC, Thermo Scientific Dionex). Thermal denaturation curves were done with Prometheus NT.48 (NanoTemper Technologies). Intrinsic fluorescence intensity at discrete wavelengths (350 and 330 nm) was measured with increasing temperature from 15 to 95 °C. T_m_ was automatically calculated by PR ThermControl. For urea-induced denaturation, monomeric DEP or Lip-DEP was incubated with various concentrations of urea for 8 h at 25 °C and analyzed by SEC-MALS. SEC-MALS was performed in Tris-buffered saline [30 mM Tris, 300 mM NaCl, NaN3 0.03% (pH 7.4)], using a GE Superdex S-200 10/300 analytical column, and analyzed as described (48).

### NMR Spectroscopy.

NMR was performed with monomeric DEP protein. NMR spectra were recorded using Bruker Avance-III spectrometers operating at ^1^H frequencies of 600 or 800 MHz, equipped with 5-mm inverse cryogenically cooled probes, and with a sample temperature of 283 K unless otherwise stated. Backbone and Cβ resonance assignments were obtained for samples with 350 µM ^13^C and ^15^N-labeled protein in an aqueous buffer containing 25 mM phosphate (pH 6.7), 150 mM sodium chloride, using standard triple resonance techniques and unmodified Bruker pulse programs. Hα resonance assignments were obtained from an HBHA(CO)NH spectrum for wt DEP and from an (H)CCH-TOCSY for the mutant, owing to difficulty in obtaining HBHA(CO)NH. Chemical shifts were referenced to the ^1^H frequency of internal 115 µM dimethylsilapentanesulfonate added to a 250 µM ^15^N-labeled sample, with X-nuclei referenced according to International Union of Pure and Applied Chemistry (IUPAC) recommendations (49). Weighted CSP in two-dimensional ^1^H-^15^N correlation, spectra was calculated as |Δδ^1^H| + |Δδ^15^N|/5, where |Δδ| is the absolute magnitude of the change in chemical shift. Steady-state {^1^H}^15^N-NOEs were acquired as pseudo–three-dimensional (3D) spectra at 800 MHz ^1^H, 64 scans per row, 256 rows, and interleaving rows with or without 120° ^1^H pulses applied at 5 ms intervals throughout the 5-s recycle delay. Samples were ^15^N-labeled at concentrations of 330 µM. ^15^N T_2_ were measured at 600 MHz ^1^H using pseudo 3D HSQC-based spectra with 2,048 in t_2_, respectively. T_2_ datasets were acquired with 12 CPMG delays from 8 to 270 ms, 16 scans, and 5 s recycle delay. H–D exchange was measured by passing 330 µM ^15^N-labeled protein through a chilled NAP-5 column (illustra) containing Sephadex G-25 resin and eluting with phosphate-buffered ^2^H_2_O. Exchange kinetics were extracted from the peak heights in BEST-TROSY spectra recorded at 9 min intervals for the first hour, then 20 min intervals for 3 h and hourly intervals up to 24 h. The dead time between buffer exchange and the start of data acquisition was 8.5 min. Per residue exchange, P_F_ were calculated from the ratio of rate constant for peak height decay to the intrinsic exchange rate obtained using the program SPHERE (50). All NMR datasets were processed using TopSpin version 3 (Bruker) and analyzed using NMRFAM-SPARKY (51). RCI-S^2^ order parameters were calculated using the program TALOS+ (37), incorporating the frequencies from Hα, H_N_, N, Cα, Cβ, and CO resonances for each residue. Where an assignment was unavailable for either wt or mutant DEP, the resonance was omitted from both assignment lists to maintain comparability.

## Supplementary Material

Supplementary File

## Data Availability

All study data are included in the article and/or *SI Appendix*.

## References

[1] M. Gammons, M. Bienz, Multiprotein complexes governing Wnt signal transduction. Curr. Opin. Cell Biol. 51, 42–49 (2018). 29153704 10.1016/j.ceb.2017.10.008

[2] C. Y. Logan, R. Nusse, The Wnt signaling pathway in development and disease. Annu. Rev. Cell Dev. Biol. 20, 781–810 (2004). 15473860 10.1146/annurev.cellbio.20.010403.113126

[3] M. Bienz, Signalosome assembly by domains undergoing dynamic head-to-tail polymerization. Trends Biochem. Sci. 39, 487–495 (2014). 25239056 10.1016/j.tibs.2014.08.006

[4] J. B. Wallingford, R. Habas, The developmental biology of dishevelled: An enigmatic protein governing cell fate and cell polarity. Development 132, 4421–4436 (2005). 16192308 10.1242/dev.02068

[5] B. T. MacDonald, K. Tamai, X. He, Wnt/beta-catenin signaling: Components, mechanisms, and diseases. Dev. Cell 17, 9–26 (2009). 19619488 10.1016/j.devcel.2009.06.016 PMC2861485

[6] M. Bienz, H. Clevers, Armadillo/beta-catenin signals in the nucleus–Proof beyond a reasonable doubt? Nat. Cell Biol. 5, 179–182 (2003). 12646868 10.1038/ncb0303-179

[7] J. D. Axelrod, J. R. Miller, J. M. Shulman, R. T. Moon, N. Perrimon, Differential recruitment of Dishevelled provides signaling specificity in the planar cell polarity and Wingless signaling pathways. Genes Dev. 12, 2610–2622 (1998). 9716412 10.1101/gad.12.16.2610 PMC317102

[8] J. B. Wallingford, S. E. Fraser, R. M. Harland, Convergent extension: The molecular control of polarized cell movement during embryonic development. Dev. Cell 2, 695–706 (2002). 12062082 10.1016/s1534-5807(02)00197-1

[9] K. Itoh, S. Y. Sokol, Axis determination by inhibition of Wnt signaling in Xenopus. Genes Dev. 13, 2328–2336 (1999). 10485853 10.1101/gad.13.17.2328 PMC316989

[10] T. Schwarz-Romond ., The DIX domain of Dishevelled confers Wnt signaling by dynamic polymerization. Nat. Struct. Mol. Biol. 14, 484–492 (2007). 17529994 10.1038/nsmb1247

[11] M. V. Gammons, M. Renko, C. M. Johnson, T. J. Rutherford, M. Bienz, Wnt signalosome assembly by DEP domain swapping of dishevelled. Mol. Cell 64, 92–104 (2016). 27692984 10.1016/j.molcel.2016.08.026 PMC5065529

[12] M. Fiedler, C. Mendoza-Topaz, T. J. Rutherford, J. Mieszczanek, M. Bienz, Dishevelled interacts with the DIX domain polymerization interface of Axin to interfere with its function in down-regulating β-catenin. Proc. Natl. Acad. Sci. U.S.A. 108, 1937–1942 (2011). 21245303 10.1073/pnas.1017063108 PMC3033301

[13] H. C. Wong ., Structural basis of the recognition of the dishevelled DEP domain in the Wnt signaling pathway. Nat. Struct. Biol. 7, 1178–1184 (2000). 11101902 10.1038/82047 PMC4381838

[14] M. J. Bennett, M. P. Schlunegger, D. Eisenberg, 3D domain swapping–A mechanism for oligomer assembly. Protein Sci. 4, 2455–2468 (1995). 8580836 10.1002/pro.5560041202 PMC2143041

[15] M. V. Gammons, T. J. Rutherford, Z. Steinhart, S. Angers, M. Bienz, Essential role of the Dishevelled DEP domain in a Wnt-dependent human-cell-based complementation assay. J. Cell Sci. 129, 3892–3902 (2016). 27744318 10.1242/jcs.195685 PMC5087658

[16] F. Rousseau, J. Schymkowitz, L. S. Itzhaki, J. Mattews, Implications of 3D domain swapping for protein folding, misfolding and function. Adv. Exp. Med. Biol. 747, 137–152 (2012). 22949116 10.1007/978-1-4614-3229-6_9

[17] H. Strutt, S. J. Warrington, D. Strutt, Dynamics of core planar polarity protein turnover and stable assembly into discrete membrane subdomains. Dev. Cell 20, 511–525 (2011). 21497763 10.1016/j.devcel.2011.03.018 PMC3094756

[18] H. Strutt, J. Gamage, D. Strutt, Robust asymmetric localization of planar polarity proteins is associated with organization into signalosome-like domains of variable stoichiometry. Cell Rep. 17, 2660–2671 (2016). 27926869 10.1016/j.celrep.2016.11.021 PMC5177602

[19] J. D. Axelrod, Unipolar membrane association of Dishevelled mediates Frizzled planar cell polarity signaling. Genes Dev. 15, 1182–1187 (2001). 11358862 10.1101/gad.890501 PMC313798

[20] U. Rothbächer ., Dishevelled phosphorylation, subcellular localization and multimerization regulate its role in early embryogenesis. EMBO J. 19, 1010–1022 (2000). 10698942 10.1093/emboj/19.5.1010 PMC305640

[21] J. Wang ., Dishevelled genes mediate a conserved mammalian PCP pathway to regulate convergent extension during neurulation. Development 133, 1767–1778 (2006). 16571627 10.1242/dev.02347 PMC4158842

[22] J. M. González-Sancho ., Functional consequences of Wnt-induced dishevelled 2 phosphorylation in canonical and noncanonical Wnt signaling. J. Biol. Chem. 288, 9428–9437 (2013). 23396967 10.1074/jbc.M112.448480 PMC3611012

[23] K. N. Schaefer, M. Peifer, Wnt/Beta-catenin signaling regulation and a role for biomolecular condensates. Dev. Cell 48, 429–444 (2019). 30782412 10.1016/j.devcel.2019.01.025 PMC6386181

[24] O. Bernatík ., Functional analysis of dishevelled-3 phosphorylation identifies distinct mechanisms driven by casein kinase 1ε and frizzled5. J. Biol. Chem. 289, 23520–23533 (2014). 24993822 10.1074/jbc.M114.590638 PMC4156093

[25] H. Strutt, M. A. Price, D. Strutt, Planar polarity is positively regulated by casein kinase Iepsilon in Drosophila. Curr. Biol. 16, 1329–1336 (2006). 16824921 10.1016/j.cub.2006.04.041

[26] H. Strutt, J. Gamage, D. Strutt, Reciprocal action of Casein Kinase Iε on core planar polarity proteins regulates clustering and asymmetric localisation. eLife 8, e45107 (2019). 31090542 10.7554/eLife.45107 PMC6542583

[27] T. J. Klein, A. Jenny, A. Djiane, M. Mlodzik, CKIepsilon/discs overgrown promotes both Wnt-Fz/beta-catenin and Fz/PCP signaling in Drosophila. Curr. Biol. 16, 1337–1343 (2006). 16824922 10.1016/j.cub.2006.06.030

[28] W. A. Yanfeng ., Functional dissection of phosphorylation of Disheveled in Drosophila. Dev. Biol. 360, 132–142 (2011). 21963539 10.1016/j.ydbio.2011.09.017 PMC3221411

[29] P. Mertins ., Proteogenomics connects somatic mutations to signalling in breast cancer. Nature 534, 55–62 (2016). 27251275 10.1038/nature18003 PMC5102256

[30] K. Hanáková ., Comparative phosphorylation map of Dishevelled 3 links phospho-signatures to biological outputs. Cell Commun. Signal. 17, 170 (2019). 31870452 10.1186/s12964-019-0470-z PMC6927192

[31] M. T. Veeman, J. D. Axelrod, R. T. Moon, A second canon. Functions and mechanisms of beta-catenin-independent Wnt signaling. Dev. Cell 5, 367–377 (2003). 12967557 10.1016/s1534-5807(03)00266-1

[32] N. Yokoyama, U. Golebiewska, H. Y. Wang, C. C. Malbon, Wnt-dependent assembly of supermolecular Dishevelled-3-based complexes. J. Cell Sci. 123, 3693–3702 (2010). 20940260 10.1242/jcs.075275 PMC2964105

[33] D. V. Tauriello ., Wnt/β-catenin signaling requires interaction of the Dishevelled DEP domain and C terminus with a discontinuous motif in Frizzled. Proc. Natl. Acad. Sci. U.S.A. 109, E812–E820 (2012). 22411803 10.1073/pnas.1114802109 PMC3325702

[34] W. J. Pan ., Characterization of function of three domains in dishevelled-1: DEP domain is responsible for membrane translocation of dishevelled-1. Cell Res. 14, 324–330 (2004). 15353129 10.1038/sj.cr.7290232

[35] I. J. Byeon, J. M. Louis, A. M. Gronenborn, A captured folding intermediate involved in dimerization and domain-swapping of GB1. J. Mol. Biol. 340, 615–625 (2004). 15210358 10.1016/j.jmb.2004.04.069

[36] D. Rogerson ., Efficient genetic encoding of phosphoserine and its nonhydrolyzable analog. Nat. Chem. Biol. 11, 496–503 (2015). 26030730 10.1038/nchembio.1823 PMC4830402

[37] Y. Shen, F. Delaglio, G. Cornilescu, A. Bax, TALOS+: A hybrid method for predicting protein backbone torsion angles from NMR chemical shifts. J. Biomol. NMR 44, 213–223 (2009). 19548092 10.1007/s10858-009-9333-z PMC2726990

[38] N. Kinoshita, H. Iioka, A. Miyakoshi, N. Ueno, PKC delta is essential for Dishevelled function in a noncanonical Wnt pathway that regulates Xenopus convergent extension movements. Genes Dev. 17, 1663–1676 (2003). 12842914 10.1101/gad.1101303 PMC196137

[39] L. C. Sheldahl ., Dishevelled activates Ca2+ flux, PKC, and CamKII in vertebrate embryos. J. Cell Biol. 161, 769–777 (2003). 12771126 10.1083/jcb.200211094 PMC2199364

[40] D. M. Velázquez, M. C. Castañeda-Patlán, M. Robles-Flores, Dishevelled stability is positively regulated by PKCζ-mediated phosphorylation induced by Wnt agonists. Cell. Signal. 35, 107–117 (2017). 28366812 10.1016/j.cellsig.2017.03.023

[41] V. Bryja, G. Schulte, N. Rawal, A. Grahn, E. Arenas, Wnt-5a induces Dishevelled phosphorylation and dopaminergic differentiation via a CK1-dependent mechanism. J. Cell Sci. 120, 586–595 (2007). 17244647 10.1242/jcs.03368

[42] F. Witte ., Negative regulation of Wnt signaling mediated by CK1-phosphorylated dishevelled via Ror2. FASEB J. 24, 2417–2426 (2010). 20215527 10.1096/fj.09-150615

[43] F. Cong, L. Schweizer, H. Varmus, Casein kinase Iepsilon modulates the signaling specificities of dishevelled. Mol. Cell. Biol. 24, 2000–2011 (2004). 14966280 10.1128/MCB.24.5.2000-2011.2004 PMC350543

[44] M. Boutros, N. Paricio, D. I. Strutt, M. Mlodzik, Dishevelled activates JNK and discriminates between JNK pathways in planar polarity and wingless signaling. Cell 94, 109–118 (1998). 9674432 10.1016/s0092-8674(00)81226-x

[45] F. Rousseau, J. W. Schymkowitz, L. S. Itzhaki, The unfolding story of three-dimensional domain swapping. Structure 11, 243–251 (2003). 12623012 10.1016/s0969-2126(03)00029-7

[46] T. Mund ., Disinhibition of the HECT E3 ubiquitin ligase WWP2 by polymerized Dishevelled. Open Biol. 5, 150185 (2015). 26701932 10.1098/rsob.150185 PMC4703060

[47] B. K. Koo ., Tumour suppressor RNF43 is a stem-cell E3 ligase that induces endocytosis of Wnt receptors. Nature 488, 665–669 (2012). 22895187 10.1038/nature11308

[48] J. Madrzak ., Ubiquitination of the Dishevelled DIX domain blocks its head-to-tail polymerization. Nat. Commun. 6, 6718 (2015). 25907794 10.1038/ncomms7718 PMC4423210

[49] J. L. Markley .; IUPAC-IUBMB-IUPAB Inter-Union Task Group on the Standardization of Data Bases of Protein and Nucleic Acid Structures Determined by NMR Spectroscopy, Recommendations for the presentation of NMR structures of proteins and nucleic acids. J. Biomol. NMR 12, 1–23 (1998). 9729785 10.1023/a:1008290618449

[50] Y.-Z. Zhang, “Protein and peptide structure and interactions studied by hydrogen exchange and NMR,” PhD thesis, Structural Biology and Molecular Biophysics, University of Pennsylvania, Philadelphia, PA (1995).

[51] W. Lee, M. Tonelli, J. L. Markley, NMRFAM-SPARKY: Enhanced software for biomolecular NMR spectroscopy. Bioinformatics 31, 1325–1327 (2015). 25505092 10.1093/bioinformatics/btu830 PMC4393527

