# Supplementary material for: Regulation of Dishevelled DEP domain swapping by conserved phosphorylation sites
Source: Proc Natl Acad Sci U S A. 2021 Jun 21;118(26):e2103258118. doi: 10.1073/pnas.2103258118 (PMC8256032; doi:10.1073/pnas.2103258118)
Supplement: Supplementary File [file pnas.2103258118.sapp.pdf]

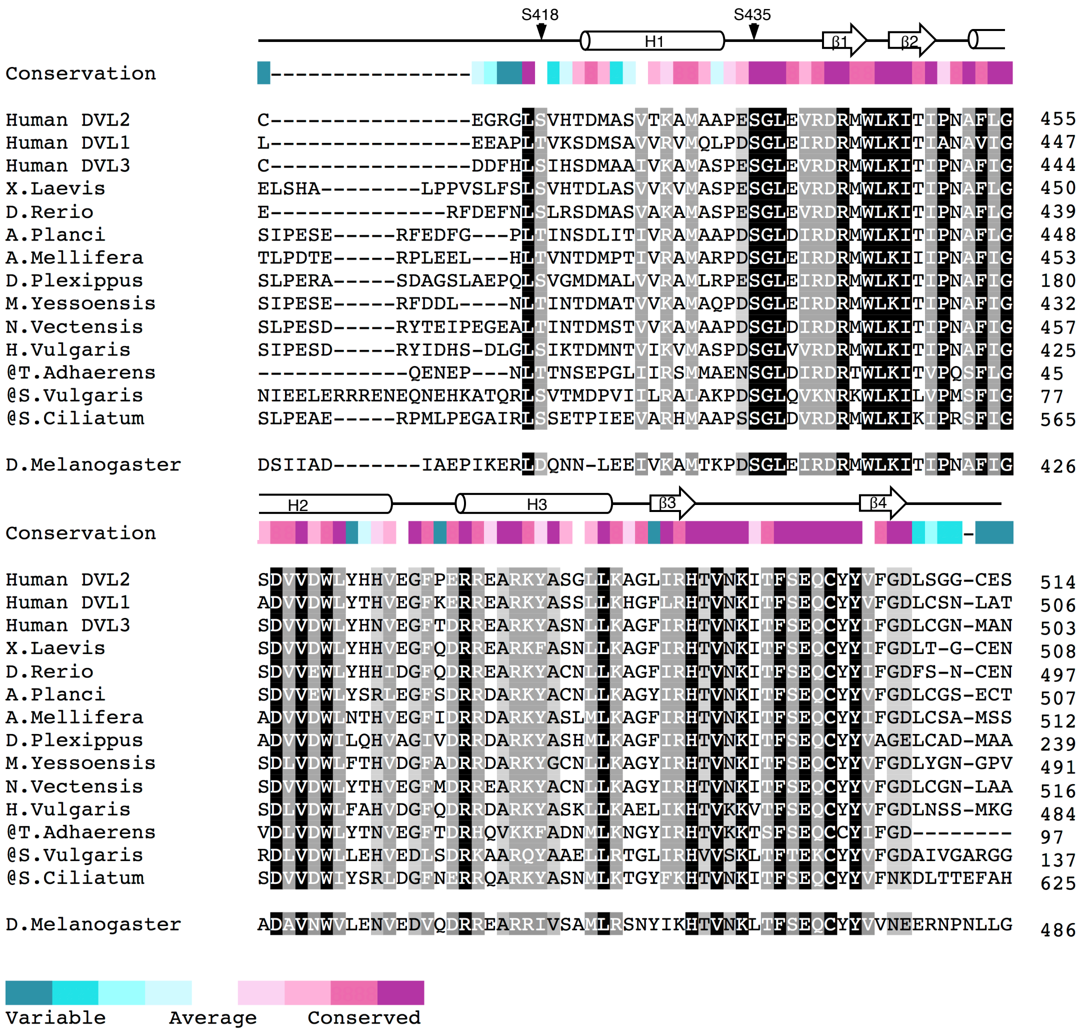

Fig. S1 (related to main **Fig. 2**)

Sequence conservation of the Dishevelled DEP domain. Sequence alignments of the DVL DEP domain across diverse animal species, @ full sequence not available. Conservation generated by multiple sequence alignment (shading) and Consurf web server (color code).

Beitia et al., Supplementary Figure 1

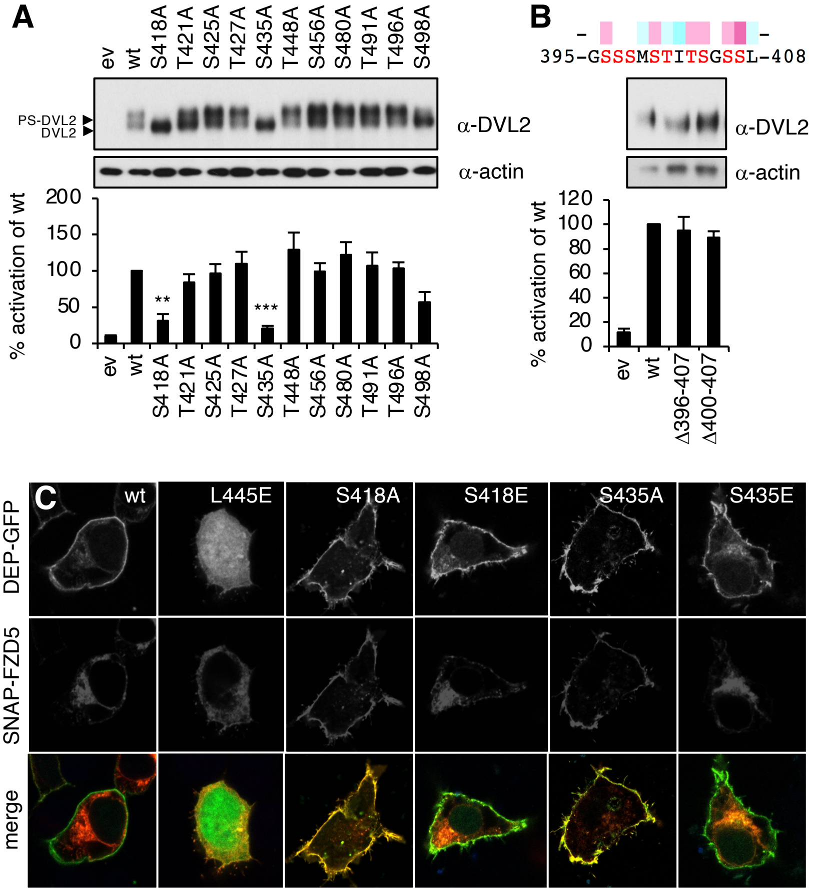

Fig. S2 (related to main **Fig. 2**)

Effects of DEP mutations on overexpressed DVL2 induced β-catenin signalling and DEP Frizzled recruitment. SuperTOP assays, monitoring the signalling activity of overexpressed hDVL2 DEP S/T>A mutants (A) or deletion of the phosphorylation cluster upstream of the DEP (B) in transiently transfected HEK293T cells (levels monitored by Western blot, *above*); error bars, SEM of >3 independent experiments; one-way ANOVA with multiple comparisons (mean of each column compared to wt control) **=p<0.01, ***=p<0.001. C) Representative images of HEK293T cells co-expressing DEP-GFP mutants and SNAP-FZD (quantitative analysis shown in **Fig. 1C**).

Beitia et al., Supplementary Figure 2

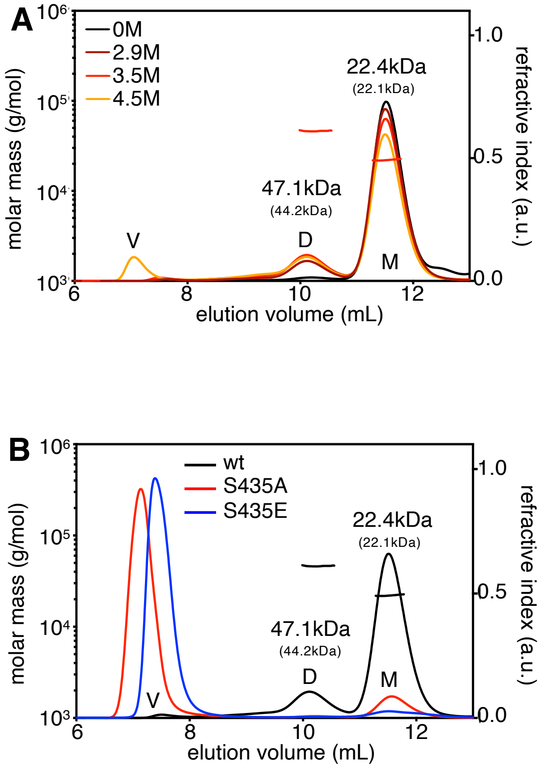

Fig. S3 (related to main **Fig. 3**)

SEC-MALS analysis of purified Lip-DEP domain. A) Elution profiles of wt Lip-DEP$_{402\text{-}510}$ monomer following incubation with urea (0M, *black;* 2.9-4.5M, *colour*) revealing monomer (M), dimer (D) and void (V). Molecular mass (MM) of M and D were determined at 3.5M urea (expected MM shown below in brackets). **B**) Elution profiles of wt (*black*) and mutant (S435A, *red*; S435E, *blue*) Lip-DEP monomer following incubation with 3.5M urea (M, D, V, and MM as in **A**).

A

amino acid	wt	ctrl	S435pS
	sample composition %		
pSer	-	-	60.4
Ser	100	11.3	8.6
Gln	-	79.3	15.8
Tyr	-	9.2	13.5
Gly	-	0.1	1.8

B

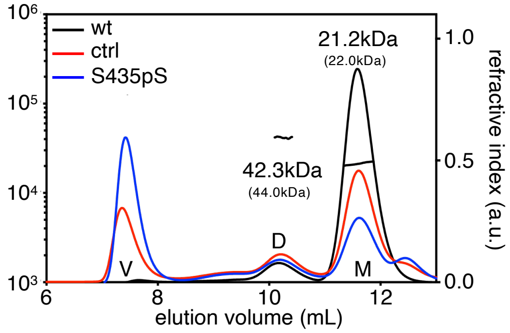

Fig. S4 (related to main **Fig. 3**)

SEC-MALS analysis of purified Lip-DEP S435pS. A) Mass Spectrometry (MS) analysis of amino acid composition of Lip-DEP$_{416\text{-}511}$ wt, ctrl and S435pS, revealing incorporation of pSer (S435pS) and misincorporation of other amino acids (ctrl and S435pS). **B**) Elution profiles of wt (*black*) ctrl (*red*) and S435pS (*blue*) Lip-DEP monomer following incubation with 3.5M urea, revealing monomer (M), dimer (D) and void (V). Molecular mass (MM) of M and D were determined for wt at 3.5M urea (expected MM shown below in brackets).

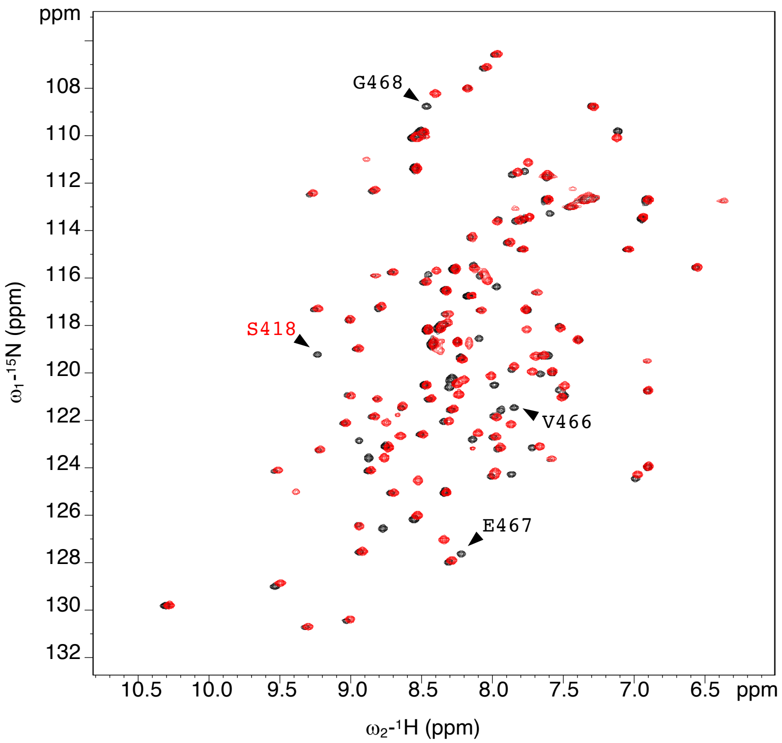

Fig. S5 (related to main **Fig. 3**)
HSQC spectra of monomeric wildtype and S418E mutant DEP. Overlay of 15N-1H correlation spectra of 350µM ^{13}C-^{15}N $DEP_{402\text{-}510}$ wildtype (black) and S418E (red); chemical shift perturbations (CSPs) are observed for H2-H3 linker residues (labeled; black).

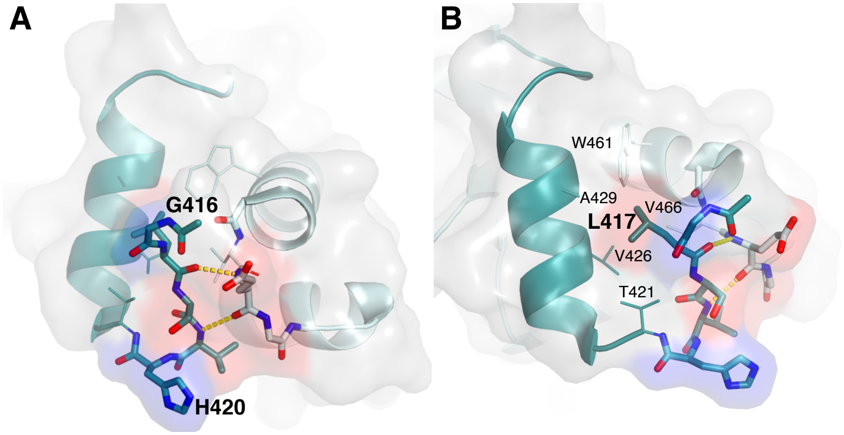

Fig. S6 (related to main **Fig. 5**)

Contacts in the domain-swapped DEP structure. (A) Partial structure of the domain-swapped DEP dimer (PDB:5SUZ); molecule A (*dark turquoise*); molecule B (*light turquoise*); *red,* residues required for intermolecular contacts; *blue*, residues not required (*labeled black in bold*) . (B) Rotated view showing L417 (*stick; labeled black in bold*) making hydrophobic contacts (*line; labeled black*).

Beitia et al., Supplementary Figure 6

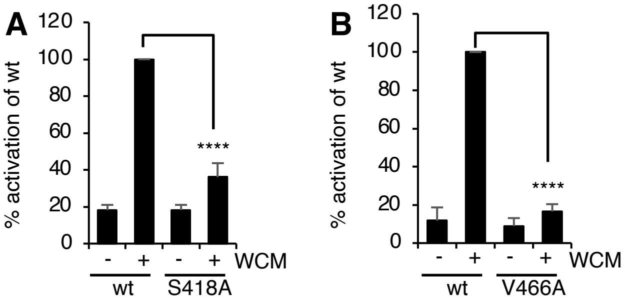

Fig. S7 (related to main **Fig. 5**)

Effects of DEP mutations on Wnt3a induced β-catenin signalling upon stable expression in DVL TKO. SuperTOP assays, monitoring the signalling activity of pBabe hDVL2-GFP stables in DVL TKO cells monitoring wt compared to S418A (A) or wt to V466A (B); WCM, 6hrs Wnt3a conditioned medium; error bars, SEM of >3 independent experiments; one-way ANOVA with multiple comparisons (mean of each column compared to wt WCM treated), ****=p<0.0001.

Beitia et al., Supplementary Figure 7

$DEP_{402-510}$	Tm(±SEM)
wt	49.1±0.4
S418A	43.1±0.5
S418E	39.7±0.4

Table. S1 (related to main **Fig. 2**)

Prometheus thermal denaturation. Cleaved $DEP_{402-510}$ wildtype (wt) and mutant (S418A/E) monomeric protein was subjected to thermal denaturation. Tm=average of three separate experiments; SEM = standard error of the mean.

mutant	DVL2 signalling activity
wt	+++
S418N	+++
S418T	++
S418H	++
S418A	+
S418E	+
S418G	+
S418L	+
S418R	+

Table. S2 (related to main **Fig. 5**)

Mutation screening of S418. Effects of S418 single amino acid substitutions on DVL2 function in transiently tracsfected HEK293T cells. Signalling activity (as measured by SuperTOP) of full-length FLAG-DVL2 bearing DEP mutation; +++, wt; ++, >50% of wt; +, <50% of wt.
